# Nanocosmetics and Skin Health: A Comprehensive Review of Nanomaterials in Cosmetic Formulations

**DOI:** 10.7759/cureus.52754

**Published:** 2024-01-22

**Authors:** Anjali S Pandey, Dushyant Bawiskar, Vasant Wagh

**Affiliations:** 1 Medicine and Surgery, Jawaharlal Nehru Medical College, Datta Meghe Institute of Higher Education and Research, Wardha, IND; 2 Sports Medicine, Abhinav Bindra Sports Medicine and Research Institute, Bhubaneswar, IND; 3 Community Medicine, Jawaharlal Nehru Medical College, Datta Meghe Institute of Higher Education and Research, Wardha, IND

**Keywords:** penetration enhancement, active compounds, nanoscale ingredients, multifunctional skincare, delivery systems, cosmetic formulations, skincare nanomaterials, nanotechnology

## Abstract

The development of nanocosmetics nanotechnology has ushered in a new age in cosmetic research, completely changing the skincare scene. This abstract investigates the relationship between skincare and nanotechnology, particularly emphasizing the effects of nanocosmetics on skin health. Cosmetics, known as "nanocosmetics," use materials at the nanoscale, typically between 1 and 100 nanometers, to improve the effectiveness and delivery of active chemicals. Nanotechnology in cosmetics allows for the development of sophisticated delivery methods that provide enhanced stability and tailored distribution, including nanoemulsions and nanocapsules. This breakthrough overcomes the constraints of conventional formulations by enabling the entry of active ingredients into the skin's deeper layers. Studies investigating nanocosmetics and skin health were included. This encompassed in vitro studies, animal models, and clinical studies of various designs. Exclusion criteria included studies focusing solely on nanotechnology unrelated to skin health or nanocosmetics and review articles editorials, commentaries, and conference abstracts. Nanocosmetics is a groundbreaking development in skincare that provides creative answers to a range of skin issues. As the area develops, realizing the full potential of nanotechnology in fostering ideal skin health will need sustained research and adherence to safety regulations.

## Introduction and background

Nanotechnology has completely transformed a number of industries, but the cosmetics industry is one where its influence is especially felt. The term "nanocosmetics" describes makeup that uses nanotechnology and materials at the nanoscale to improve skincare formulation performance, distribution, and efficacy. With the introduction of nanotechnology into cosmetics, a new era of sophisticated skincare products that target different skin issues and enhance general skin health has begun. Ingredients used in nanocosmetics are often measured at the nanoscale, which is between 1 and 100 nanometers [1]. This makes it possible for chemicals to penetrate the skin more deeply and reach layers that may be difficult for regular substances to reach. Advanced delivery methods, including nanoemulsions, nanocapsules, and nanosomes, are made possible by nanotechnology. To ensure that active chemicals reach the desired skin layers for optimal efficacy, these methods improve the stability and targeted administration of the substances. Better penetration through the skin barrier is made possible by the smaller particle sizes of the nanomaterials used in cosmetics. Improved bioavailability of active ingredients may arise from this, resulting in skincare effects that are more pronounced and long-lasting. Specific skincare requirements can be addressed by tailoring nanocosmetic compositions. Because of their adaptability, nanoparticles may be used to create customized solutions that address a variety of problems, including pigmentation, hydration, aging, and more. Nanocosmetics frequently incorporate several uses with one product. These multipurpose formulas offer complete skincare treatments by combining anti-aging agents, antioxidants, UV protection, and moisturizing elements [2].

## Review

Search methodology

The methodology involves a comprehensive literature search strategy using multiple electronic databases including PubMed, Scopus, and Google Scholar. The search terms used were related to “nanotechnology,” “cosmetic formulations,” “multifunctional skincare,” and “active compounds.” In addition to electronic database searches, the reference lists of relevant articles and review papers were manually searched for additional studies. No language restrictions were applied, but only studies published up to the current knowledge to date were included. The inclusion criteria were defined to select studies that were relevant and of high quality. Studies investigating nanocosmetics and skin health were included. This encompassed in vitro studies, animal models, and clinical studies of various designs. Exclusion criteria included studies focusing solely on nanotechnology unrelated to skin health or nanocosmetics and review articles, editorials, commentaries, and conference abstracts. Two independent reviewers screened the title and abstracts, followed by a full-text assessment of selected articles. Disagreements were resolved through consensus or consultation with a third reviewer if needed. The methodology aimed to include high-quality studies that contributed to the comprehensive understanding of nanocosmetics and skin health. By employing a rigorous search strategy and applying strict inclusion and exclusion criteria, a robust selection of studies was identified to inform the review. Editor’s note was also filtered out (Table 1)

**Table 1:** List of studies included in the review

Author	Year	Type	Conclusion
Mavridi-Printezi et al. [3]	2020	Randomized controlled review	The exceptional plasticity of both natural and artificial melanin for the construction of multifunctional nanoplatforms is revealed by recent advancements in the bioapplication of melanin nanoparticles to nanomedicine and, more recently, to nanocosmetics. As was extensively covered in the preceding sections, these bioinspired and inherently biocompatible materials have an extraordinary range of features.
Vogel et al. [4]	2021	Original randomized controlled trials	Aside from knowing how to avoid its triggering factors, it is crucial to treat the changes in the set of degenerative and progressive effects that occur in the subcutaneous tissue and skin texture. This variation should be considered a pathology rather than the appearance of unattractive skin.
Lee et al. [5]	2020	Original article	According to the current study, there may be a risk of damage from using sunscreens containing nanoparticles, which might lead to greater urine 8-hydroxy-2'-deoxyguanosine levels. The risk of skin cancer is decreased by sunscreen usage, which much surpasses any possible long-term hazards. But one cannot disregard the exposure risk associated with wearing certain cosmetics.
Ditlopo et al. [6]	2022	Original article	The effectiveness of *Hoodia gordonii* natural extract as a catalyst for the production of single phase CeO2 nanocrystals was confirmed by this work. These bioengineered nanocrystals demonstrated an extraordinary optical selectivity for UV filtering that was almost identical to that of an ideal UV filtering system.
Raszewska-Famielec and Flieger [7]	2022	Open access randomized controlled trials	Interest in the harmful consequences of nanomaterial exposure on people and the environment has developed as a result of its application in cosmetic and medicinal goods. The majority of focus is on metal oxide nanoparticles (NPs), which are frequently included to photoprotective cosmetics. The ecology may be seriously threatened by the discharge of metallic NPs into the environment.

Nanocosmetics

Cosmetics that use nanotechnology, which entails modifying materials at the nanoscale, usually with dimensions smaller than 100 nanometers, are referred to as nanocosmetics. With the use of this technology, incredibly small particles with special qualities and possible advantages in cosmetics may be created and used. The tiny size of the particles employed in nanocosmetics is its distinguishing characteristic. Cosmetics commonly include nanoparticles with sizes between 1 and 100 nanometers. In cosmetic compositions, its smaller size enables better texture, simpler absorption, and better performance. Smaller particles have a greater ability to permeate the skin than bigger ones. This feature is used to enhance the active ingredient's distribution, enabling improved absorption and possible effectiveness. Cosmetic products with a smoother texture may benefit from the use of nanoparticles. They can also improve a formulation's transparency, which improves its visual appeal and user comfort. Certain compounds may have better stability, thanks to nanocosmetics [8].

Cosmetic items' shelf lives can be increased by using nanoparticles to assist stop the breakdown of active ingredients. Sunscreens with transparent formulations that effectively block UV rays are frequently made using nanotechnology. It is possible to employ nanoparticles, such as zinc oxide or titanium dioxide, which provide broad-spectrum UV protection without the white cast that comes with bigger particles. Antioxidants may be efficiently encapsulated and delivered by a nanoparticle design. This method of tailored distribution has the potential to improve cosmetic goods' antioxidant qualities. Targeted distribution of active chemicals to certain skin layers or cells is made possible by nanotechnology. This focused approach could improve skincare products' efficacy. Although there are distinct benefits to using nanocosmetics, there are worries over the safety of nanoparticles. The tiny size begs the issues of possible systemic absorption and penetration into deeper epidermal layers. The safety of chemicals found in nanocosmetics is still being investigated. Global regulatory bodies are currently reviewing and developing policies on the use of nanoparticles in cosmetics. Adherence to rules is crucial in guaranteeing the safety and effectiveness of nanocosmetic products [9].

Cosmetic formulation

Carefully combining different substances to produce a product that improves or preserves the look of the skin, hair, nails, or general beauty is known as cosmetic formulation. For these compositions to be effective, stable, and provide the customer with a pleasing sensory experience, science, technology, and creativity are combined.

The elements that provide a cosmetic product its main advantages are called active ingredients. These can contain vitamins, peptides, antioxidants, moisturizers, and other compounds with particular skincare or beauty-enhancing qualities. The texture, consistency, and general structure of cosmetic compositions are derived from base components. Oils, waxes, emulsifiers, and water are examples of common basic components. In order to formulate goods that blend oil and water components to create stable emulsions, emulsifiers are necessary. For many creams and lotions used in healthcare and cosmetic items, this is essential. In order to stop bacteria, molds, and other microbes from growing, preservatives are added to cosmetic compositions. They contribute to the product's increased shelf life and continued safety throughout its usage [3].

In order to improve the sensory experience of utilizing cosmetic goods, fragrances are frequently incorporated. They might arouse favorable feelings and add to the overall charm. A variety of substances, including gelling agents, stabilizers, and thickeners, are used to change the consistency and texture of cosmetic products. These guarantee a user-friendly application and experience. In order to give cosmetic items a particular color, colorants are added. They are frequently employed to produce desired esthetic effects in hair dyes, nail paints, and cosmetics. Humectants aid in hydrating the skin by drawing in and holding onto moisture. Propylene glycol, hyaluronic acid, and glycerin are examples of common humectants [4].

Cosmetic compositions' acidity or alkalinity can be adjusted with pH adjusters. Ensuring that the product remains stable and functional necessitates maintaining the proper pH level. To offer protection against UV radiation, sunscreen agents may be added to skincare products, particularly those intended for use during the day. Certain formulas contain specialty components that address certain issues, such as brightening agents, acne-fighting compounds, or anti-aging peptides. The use of sustainable and ethical elements in cosmetic formulations is becoming more popular, which is in line with the customer's desire for materials that are obtained ethically and environmentally. Cosmetic formulas are put through a thorough testing process to ensure stability, effectiveness, and safety. This covers testing for dermatology, microbiological testing, and stability testing under various circumstances [10].

A major development in cosmetic science is the addition of nanomaterials to cosmetic compositions. Materials may be manipulated at the nanoscale, which is often measured in nanometers, thanks to nanotechnology. This has created new opportunities to improve the cosmetic products' overall efficacy, performance, and delivery.

These minerals' nanoparticles are utilized in sunscreens to offer broad-spectrum UV protection. The smaller size eliminates the conventional problem of a white cast linked to bigger particles and permits transparency on the skin. Active chemicals can be encapsulated and delivered via nanosized vesicles called liposomes and nanosomes, which improved stability, targeted distribution, and penetration of active ingredients. Colloidal dispersions with droplet sizes in the nanometer range are called nanoemulsions, which expanded the surface area, improved stability, and improved oil and active ingredient absorption in skincare products. Enclosing active substances in nanocarriers to prevent degradation and provide regulated release is known as nanoencapsulation, which extended effectiveness, improved stability, and focused distribution of active ingredients [11].

Nanoparticles in sunscreen

Sunscreen nanoparticles are essential for improving the performance, look, and feel of sun protection solutions. These nanoparticles are designed to be at the nanoscale; commonly, they are zinc oxide and titanium dioxide. Because titanium dioxide and zinc oxide nanoparticles are smaller than the typical particles, transparency is increased. This takes care of the cosmetic issue of the skin appearing white or chalky, which was linked to older, bigger particles. Because the nanoparticles are smaller, the texture of the sunscreen is smoother, which makes it easier to apply and gives consumers a more enjoyable sensory experience. Anti-UVA and UVB radiation is effectively defended against by nanoparticles. Because of their larger surface area, nanoparticles are better able to disperse and absorb dangerous UV rays, which lowers the risk [1].

Specialized delivery methods like liposomes and nanosomes are employed in skincare and cosmetic formulations. The transport and effectiveness of active substances are improved by the special qualities of these nanoscale structures. Liposomes are lipid bilayers, one or more of which form spherical vesicles. They unite to create a closed lipid membrane, forming an enclosed area capable of transporting molecules that are soluble in lipids and water. Phospholipids, which resemble the lipids in cell membranes, are commonly used to make liposomes. These phospholipids' hydrophilic (which attracts water) and hydrophobic (which repels water) portions support the liposome's structure. Liposomes serve as active ingredient delivery systems or carriers. Their shape allows them to encapsulate and transport both hydrophilic and hydrophobic molecules, preserving them from degradation and enhancing their bioavailability [6]. Like liposomes, nanosomes are nanosized vesicles that are particularly made to penetrate skin more deeply. Similar to liposomes, they are composed of a lipid bilayer. Lipids also make up nanosomes, and different lipid components may be used in their creation. These lipids support the shape and operation of the nanosomes [12]. Although they are designed for better skin penetration, nanosomes behave similarly to liposomes in terms of distribution. They are especially helpful in providing a more focused and effective administration of active substances by getting them into the skin's deeper layers. Because of their tiny size, nanosomes may more easily penetrate the epidermis and reach places that bigger particles might find difficult to reach. This helps skincare products work more effectively by distributing active ingredients exactly where they are required [13].

Serums, creams, and lotions are examples of skincare products that frequently contain liposomes or nanosomes. They are used to provide a variety of active components, such as peptides, vitamins, antioxidants, and moisturizing agents. These nanoscale structures are used in the creation of sophisticated skincare solutions that address certain issues including skin restoration, hydration, and anti-aging [14].

Nutricosmetics

A combination of the words "nutrition" and "cosmetics," "nutricosmetics" refers to oral skincare and cosmetic items that improve appearance and support healthy skin. Bioactive components like vitamins, minerals, antioxidants, and other nutrients with alleged advantages for skin, hair, and nails are commonly found in these products. Nutricosmetics are ingested orally, usually as functional meals, drinks, or supplements. Their purpose is to nourish the skin from the inside out, in harmony with topical skincare products. Antioxidants like as polyphenols, beta-carotene, and vitamins C and E are frequently used in nutricosmetics to scavenge free radicals and guard against oxidative stress. Supplements containing collagen are often used to promote skin suppleness and lessen the visibility of wrinkles and fine lines. Some of the nutrients that are included include biotin, zinc, vitamin A, and vitamin D because of their involvement in nail strength, hair development, and skin health. Omega-3 fatty acids are frequently obtained from plant or fish oil sources, and their potential benefits in preserving skin moisture and suppleness may warrant their inclusion [15].

Biotin, also referred to as vitamin B7 or vitamin H, is essential for maintaining the health of the skin, hair, and nails. This vitamin, which belongs to the B complex family of vitamins and is water-soluble, is necessary for a number of body processes, including the upkeep of healthy skin, hair, and nails. Biotin has a role in keeping the skin cells in a healthy state. It aids in the synthesis of fatty acids, which are necessary for keeping the skin healthy. Biotin is frequently linked to enhancing hair health and development. It contributes to the synthesis of keratin, a protein that gives hair its structure. Strong and healthy nails are thought to be maintained in part by biotin [16]. Zinc is an essential element that supports healthy skin, hair, and nails among other physiological processes. Because of its participation in several enzymatic activities, cell division, and tissue development, it makes a substantial contribution to these fields. Zinc supports the activity of enzymes involved in DNA synthesis and repair, which helps to maintain healthy skin. It possesses antioxidant qualities that can guard against harm from free radicals, aids in wound healing, and helps control inflammation. Proper hair growth and repair require zinc. It contributes to the creation of many proteins, including keratin, which is essential to the structural integrity of the hair. Zinc has a role in tissue growth and development as well as the synthesis of proteins [17]. Fat-soluble vitamin A is essential for keeping the skin, hair, and nails in good condition. Because it is involved in a number of physiological processes, it makes significant contributions to these domains. For the preservation and health of the skin, vitamin A is necessary. It aids in the development and maintenance of skin tissues, controls the growth and division of skin cells, and aids in the synthesis of collagen, a protein that gives the skin its structure. Hair follicle development and health are supported by vitamin A. It helps the scalp produce sebum, an oily material that hydrates the scalp and maintains the health of hair strands. Nail strength and development are facilitated by vitamin A. It aids in the keratinocytes' growth [18]. It is well recognized that vitamin D supports immune system function, bone health, and the control of cell proliferation, among other aspects of general health. The skin starts to produce vitamin D when it is exposed to sunshine. Sufficient amounts of vitamin D promote healthy skin by lowering inflammation, promoting wound healing, and maybe helping to treat skin diseases like psoriasis. Hair follicles have vitamin D receptors, and it is thought that vitamin D regulates the cycles of hair development. A small body of studies indicates that vitamin D could help promote normal nail development.

Nanoemulsions

Because of their special qualities and possible advantages, colloidal dispersions with droplet sizes in the nanometer range, known as nanoemulsions, have drawn interest in the cosmetic industry. Tiny droplets with a vast surface area make up nanoemulsions [19]. This feature makes it possible for active substances to penetrate the skin more effectively. Therefore, skincare products that include nanoemulsions may improve the way that healthy ingredients are absorbed. Formulations for skincare are more stable, thanks to nanoemulsions. Phase separation and the deterioration of active substances are less likely due to the tiny droplet size. This stability gives the product a longer shelf life by ensuring that it keeps its effectiveness throughout time. The smoother texture and enhanced attractiveness of skincare products are a result of nanoemulsions' tiny droplet size. Users' whole sensory experience is improved by this [20].

Nanoemulsions have the potential to enable the regulated release of active components. A more consistent skincare experience is made possible by the controlled release method, which enables a continuous and longer administration of therapeutic chemicals. It is possible to construct nanoemulsions with moisturizing and hydrating components. These compositions' tiny droplet sizes facilitate effective absorption and distribution, promoting skin hydration and moisture balance. Highly adaptable, lipophilic (oil-soluble) and hydrophilic (water-soluble) substances may both be encapsulated in nanoemulsions. With this dual capacity, a variety of active ingredients may be delivered to meet a range of skincare demands. Skincare products are more widely accepted as cosmetics, thanks in part to nanoemulsions. Formulations become more user-friendly due to their enhanced texture, spreadability, and general elegance, which promotes consistent usage and adherence to skincare regimens [7].

It is possible to create nanoemulsions that specifically distribute particular active substances to the appropriate layers of the skin. By treating certain skincare issues, this customized technique enables a more concentrated and effective administration of chemicals. Sunscreen compositions frequently contain nanoemulsions. Smaller droplet sizes aid in the creation of transparent formulations, which solves the esthetic problem of a white cast brought on by bigger particles seen in conventional sunscreens. It is possible to create nanoemulsions containing antioxidant and anti-aging components. These formulations' improved penetration enables them to reach deeper skin layers, which may have more significant benefits in preventing oxidative stress and lowering indications of aging [21].

Nanoencapsulation

In the realm of cosmetics, nanoencapsulation, a technique that entails encapsulating active substances within nanocarriers, has demonstrated potential for enhancing the transport, stability, and efficacy of numerous compounds [22]. The transfer of active substances to the skin's deeper layers is facilitated by nanoencapsulation. Because they are smaller than bigger particles, nanocarriers can penetrate regions that may be difficult for them to reach. Degradation may occur in active substances, particularly those that are light-, air-, or heat-sensitive. By enveloping these components in a protective shell made of nanoparticles, these substances are better stabilized and protected from outside influences. The release of active chemicals can be regulated and prolonged using nanoencapsulation. A longer lasting and more reliable distribution of the active substances is ensured by this regulated release method, which also helps to maintain the skincare effect [23].

In cosmetics, nanoencapsulation is the process of encasing active substances in small particles known as nanoparticles using nanotechnology. When added to cosmetic compositions, these particles, which are usually in the nanometer range (1-1000 nanometers), provide a number of advantages. When delicate or unstable cosmetic chemicals are exposed to light, oxygen, or heat, nanoencapsulation helps prevent their destruction. The shelf life and effectiveness of the active substances are improved by this preservation. Controlled release and targeted delivery of active substances to certain skin layers or cells are made possible by nanoparticles. This guarantees a longer-lasting and more efficient release of the active ingredients, increasing their bioavailability and effectiveness. Both hydrophilic (water soluble) and hydrophobic (oil soluble) chemicals can be encapsulated using nanoencapsulation, providing a wider range of compatibility and efficacy in cosmetic compositions [24].

Antioxidants are shielded from deterioration by light and oxygen when they are contained in nanocarriers. This guarantees that antioxidants stay powerful and offer good defense against oxidative stress, which is connected to early aging. You can use nanoencapsulation to compounds that are moisturizing and hydrating. This improves the way these chemicals are delivered, hydrating the skin and preserving its moisture balance over time. Certain compounds can be made more bioavailable via encapsulation, which increases the amount of the active component that reaches the skin's target location. This is especially crucial for maximizing skincare products' efficacy. Targeted distribution of certain active substances to the desired skin layers is made possible by nanoencapsulation. The active chemicals are guaranteed to reach the intended location of action according to this focused strategy [25].

Certain active compounds, particularly at greater concentrations, have the potential to irritate the skin. By limiting direct contact with the skin and enabling a controlled release of the active ingredient, nanoencapsulation can help lessen discomfort. Sunscreen compositions frequently use nanoencapsulation. Encapsulating active sunscreen ingredients, including UV filters, can improve their stability and efficacy and improve protection from UV radiation. Anti-aging substances like retinoids or peptides can be more effectively delivered and efficaciously when they are nonencapsulated. Increased collagen synthesis, fewer wrinkles, and a general improvement in skin texture can result from this. Customized formulations with various active component combinations may be made, thanks to nanoencapsulation. This makes it possible to create skincare solutions with several uses that are designed to address different skin issues at once [26-28].

Fullerenes and nanodiamonds in skin health

Advanced nanomaterials like fullerenes and nanodiamonds are being investigated for their uses in cosmetics and skin health. These nanoparticles are being studied for their special qualities and possible advantages in treating different skin issues; however, research is still under progress. All-carbon molecules organized in a spherical, ellipsoidal, or cylindrical shape are known as fullerenes. Carbon-60 (C60), also referred to as buckyball, is the most well-known fullerene. It is well-known that fullerenes have potent antioxidant qualities. Free radicals, which are linked to aging and environmental causes that cause skin damage, can be successfully neutralized by them. According to some research, fullerenes may provide UV protection, assisting in the reduction of UV-induced skin damage and early aging [29].

Given that fullerenes have demonstrated some anti-inflammatory properties, they may be useful in treating inflammatory skin disorders. Carbon atoms organized in a diamond lattice pattern make up nanodiamonds, which are particles at the nanoscale. Since they are commonly accepted by biological systems, nanodiamonds are regarded as biocompatible. The use of nanodiamonds as medication delivery vehicles has been investigated. They can be used in skincare products to efficiently apply active compounds to the skin. Because of their tiny size, nanodiamonds may make it easier for active substances to enter the skin, which might improve the effectiveness of skincare products. Nanodiamonds may function as a mild exfoliator in some formulations, aiding in the removal of dead skin cells and fostering skin regeneration [30].

The possibility of using fullerenes and nanodiamonds to lessen aging indicators like wrinkles and fine lines is being investigated. By reducing oxidative stress and addressing variables that contribute to uneven skin tone, fullerenes may help brighten the skin. Because of their biocompatibility, nanodiamonds may be used to aid in tissue regeneration and wound healing [31].

Nanopeptides in skin health

Smaller versions of conventional peptides that have been created at the nanoscale for improved skin absorption and distribution are known as nanopeptides. Peptides are generally short sequences of amino acids that are essential to many biological processes, including the health of the skin. In particular, nanopeptides are made to maximize the advantages of peptides for skincare applications. These peptides' nanoscale size makes for improved skin penetration. Because they may more readily cross the epidermal barrier, nanopeptides can act on deeper layers of the skin. The ability of peptides, particularly nanopeptides, to promote the production of collagen is well established. A crucial structural component in the skin that gives it suppleness and firmness is collagen [32].

Reduced oxidative stress and halted collagen deterioration are two possible anti-aging effects of nanopeptides. When combined, these benefits can give the complexion a more radiant, young appearance. Some nanopeptides could be made to draw and hold moisture in order to improve skin hydration. This may lead to softer, more youthful-looking skin and less dryness. Antioxidant qualities in nanopeptides may aid in the neutralization of free radicals, which are known to cause skin damage and premature aging. Antioxidants are essential for protecting the skin from harmful environmental factors. Peptides, including nanopeptides, may aid in the processes of cellular regeneration and repair. This may help to preserve the general health of your skin and give you a more youthful appearance [33-35].

Certain peptides have anti-inflammatory qualities, and nanopeptides with these characteristics could aid in lowering skin inflammation. Those with sensitive or inflamed skin may find this very helpful. Targeted delivery of nanopeptides enables them to target certain skin issues or reach particular layers of the skin. This focused approach improves skincare formulas' accuracy and efficacy. Because of their formulation adaptability, nanopeptides may be used to create customized skincare solutions that are aimed at specific skincare requirements. This flexibility may result in skincare products that are more specialized and customized. In order to develop comprehensive skincare formulations, nanopeptides can be mixed with additional active substances. Their ability to work with a variety of substances enables the creation of goods with many uses [36,37].

## Conclusions

Active substances can be delivered in a more focused and regulated way, thanks to nanoencapsulation. Targeting certain skin problems or concerns precisely is made possible by nanoparticles. Ingredients can be targeted to specific skin layers or cells by nanoencapsulation, allowing for a more focused therapy of conditions including wrinkles, fine lines, pigmentation, or dehydration. Ingredients that are unstable or sensitive are shielded against deterioration by light, oxygen, and temperature changes through the use of nanoencapsulation. Because they are smaller than the bigger particles present in standard formulations, nanoparticles can penetrate deeper into the skin. Incompatible or poorly soluble substances that would not function well together in conventional formulations can now be combined, thanks to nanoencapsulation. Overall, compared to conventional formulations, nanocosmetics provide a more efficient and focused approach to skincare, thanks to their improved stability and sophisticated delivery mechanisms.
